# Identification of key genes and miRNAs markers of papillary thyroid cancer

**DOI:** 10.1186/s40659-018-0188-1

**Published:** 2018-11-10

**Authors:** Jie Qiu, Wenwei Zhang, Chuanshan Zang, Xiaomin Liu, Fuxue Liu, Ruifeng Ge, Yan Sun, Qingsheng Xia

**Affiliations:** 1 Otolaryngology Head and Neck Surgery, The Affiliated Hospital of Qingdao University, No. 16 Jiangsu Road, Qingdao, 266071 Shandong Province China; 2 Radiology Department, The Affiliated Hospital of Qingdao University, Qingdao, 266003 Shandong China; 3 Otolaryngology Head and Neck Surgery, Shaoxing Municipal Hospital, Shaoxing, 312000 Zhejiang, China; 4 Otolaryngology Head and Neck Surgery, Qingdao Municipal Hospital, No. 5 Donghai Road, Qingdao, 266071 Shandong China

**Keywords:** Papillary thyroid cancer, Gene, miRNA, Tumor stage, Prognosis

## Abstract

**Objective:**

In this study, crucial genes and microRNAs (miRNAs) associated with the progression, staging, and prognosis of papillary thyroid cancer (PTC) were identified.

**Methods:**

Four PTC datasets, including our own mRNA-sequencing (mRNA-seq) dataset and three public datasets downloaded from Gene Expression Omnibus and The Cancer Genome Atlas, were used to analyze differentially expressed genes (DEGs) and miRNAs (DEMs) between PTC tumor tissues and paired normal tissues (control). Gene ontology (GO) terms and pathways associated with these DEGs were identified, and protein–protein interactions (PPIs) were analyzed. Additionally, an miRNA-mRNA regulatory network was constructed and the functions of DEMs were explored. Finally, miRNAs/mRNAs associated with tumor staging and prognosis were identified. The expression levels of several key genes and miRNAs were validated by qRT-PCR.

**Results:**

Numerous DEGs and DEMs were identified between tumor and control groups in four datasets. The DEGs were significantly enriched in cell adhesion and cancer-related GO terms and pathways. In the constructed PPI network, ITGA2, FN1, ICAM1, TIMP1 and CDH2 were hub proteins. In the miRNA-mRNA negative regulatory networks, miR-204-5p regulated the largest number of target genes, such as *TNFRSF12A*. miR-146b, miR-204, miR-7-2, and *FN1* were associated with tumor stage in PTC, and *TNFRSF12A* and *CLDN1* were related to prognosis.

**Conclusions:**

Our results suggested the important roles of *ITGA2*, *FN1*, *ICAM1*, *TIMP1* and *CDH2* in the progression of PTC. miR-204-5p, miR-7-2, and miR-146b are potential biomarkers for PTC staging and *FN1*, *CLDN1*, and *TNFRSF12A* may serve as markers of prognosis in PTC.

## Background

Thyroid cancer is the most common endocrine neoplasm, accounting for approximately 1.7% of all human malignancies [1]. Thyroid cancer is usually classified into four types: papillary thyroid cancer (PTC), anaplastic thyroid cancer, follicular thyroid cancer, and medullary thyroid cancer [2]. Among the four types, PTC is the most common, accounting for 75–85% of all thyroid cancer cases [3]. PTC is usually curable and has a 5-year survival rate of over 95% [4]. However, PTC occasionally dedifferentiates into more aggressive and lethal thyroid cancers. Additionally, recurrences are observed in approximately 30% of patients with PTC. Therefore, further analyses of the molecular characteristics of this disease are necessary [5].

Genetic mutations have been identified in the majority of PTC cases and are believed to be responsible for PTC initiation [6]. A high frequency of activating somatic alterations in genes encoding effectors in the MAPK (mitogen-activated protein kinase) signaling pathway, such as *BRAF*, *RAS*, and *RET*, has been found in PTC [7–9]; these mutations act as drivers in approximately 70% of PTC cases [10]. Additionally, galectin-3 (*LGALS3*), platelet-derived growth factor (*PDGF*), and epithelial muctin-1 (*MUC*-*1*) are also potential biomarkers of PTC [11, 12]. A recent study has demonstrated that numerous microRNAs (miRNAs) are transcriptionally up-regulated in PTC compared with normal tissues [13]. miR-181b, miR-221, and miR-222 have been suggested to be overexpressed in most thyroid tissues originating from patients affected by PTC [14]. Although numerous molecular markers associated with the carcinogenesis of PTC have been identified, significant uncertainty remains regarding its molecular mechanisms. Furthermore, prognosis- or staging-related molecular targets need to be explored.

Microarrays have made it possible to analyze genome-wide miRNA or mRNA expression [15]. In this study, we combined our mRNA-sequencing (mRNA-seq) data with three public datasets (miRNA-seq and mRNA-seq) downloaded from Gene Expression Omnibus (GEO, http://www.ncbi.nlm.nih.gov/geo/) and The Cancer Genome Atlas (TCGA, https://tcga-data.nci.nih.gov/) to analyze differentially expressed genes (DEGs) and miRNAs (DEMs) between PTC tumor tissues and normal tissue samples (control). Common miRNAs and mRNAs between our mRNA-seq dataset and public datasets were selected for subsequent analyses, including analyses of gene ontology (GO) biological processes, pathway enrichment, and protein–protein interactions (PPIs), the construction of miRNA-mRNA regulatory networks, as well as the identification of miRNAs and mRNAs associated with staging and prognosis. Moreover, the expression levels of several key mRNAs and miRNAs identified in these analyses were confirmed by qRT-PCR. Loci identified in this study may act as important therapeutic targets or prognostic markers in PTC.

## Methods

### Tissue samples and mRNA-seq data collection

Five PTC tumor samples (P1–P5) and paired peritumoral thyroid tissue samples as controls (N1–N5) were obtained from patients with PTC who underwent thyroidectomy. The tumor samples and paired peritumoral thyroid tissue samples were obtained by thyroidectomy and stored in RNA store Reagent DP408-02 (Tiangen Biotech Co., Ltd., Beijing, China) at 4 °C in a refrigerator. Tumor stages were determined based on the tumor-node-metastasis (TNM) criteria. Detailed patient information is provided in Table 1. Informed consent was obtained from all patients. This study was approved by the local Research Ethics Committee.

**Table 1:** Characteristics of patients

Samples	Hospitalization starting time	Sex	Age	TNM stage	Clinical stage
P1/N1	2014/7/31	Male	26	T1N1aM0	I
P2/N2	2014/8/13	Male	43	T1N1aM0	I
P3/N3	2014/8/11	Female	47	T1N1aM0	III
P4/N4	2014/8/11	Male	25	T1N1aM0	I
P5/N5	2014/8/12	Female	46	T1N1aM0	III
P6/N6	2017/7/27	Female	45	T1N1aM0	I
P7/N7	2017/7/27	Female	61	T1N1aM0	I
P8/N8	2017/8/1	Male	55	T1N1aM0	I
P9N9	2017/8/16	Female	50	T1N1aM0	I
P10/N10	2017/8/17	Female	45	T1N1aM0	I
P11/N11	2017/8/17	Female	66	T1N1aM0	I
P12/N12	2017/9/14	Male	33	T1N1aM0	I
P13N13	2017/9/14	Female	47	T1N1aM0	I
P14/N14	2017/9/18	Female	54	T1N1aM0	I

*P* papillary thyroid carcinoma, *N* paired adjacent normal tissues, *TNM* tumor-node-metastasis

After tissue collection, total RNAs were extracted using the RNAprep Pure Tissue Kit (Tiangen Biotech Co., Ltd.) according to the manufacturer’s protocol and was then converted to Tru-Seq libraries for sequencing using the Illumina HiSeq2000 platform (Illumina, San Diego, CA, USA). After sequencing, RNA-seq data were preprocessed, including the removal of the adaptor sequence from raw reads and the filtering of reads with a high N content and low quality. The detailed sequencing and preprocessing procedures are described in our previous study [16].

### Public data collection

miRNA microarray data (accession GSE57780) were downloaded from the GEO database. A total of 9 samples were available, i.e., 3 PTC tissues, 3 normal tissues, and 3 nodal metastases. In this study, 3 PTC and 3 normal tissues were selected for further analysis. The platform used to obtain these data was the Illumina HiSeq2000.

Additionally, PTC-associated mRNA-seq, miRNA-seq, and clinical data were downloaded from TCGA using the TCGAbiolinks R package. In total, 552 samples were included.

### Differential expression analysis

Using our own mRNA-seq data, gene expression levels were calculated using the RPKM method. DEGs between the PTC and control groups were detected using the edgeR [17] tool (version 3.4, http://www.bioconductor.org/packages/release/bioc/html/edgeR.html), and the significance of DEGs was calculated based on the negative binomial model. The obtained P-values were adjusted using the Benjamini and Hochberg method [5] to obtain the false discovery rate (FDR). The thresholds for DEG selection were FDR < 0.05 and |log2 fold change (FC)| > 2.

The mRNA-seq and miRNA-seq data obtained from TCGA and GEO were also preprocessed using edgeR [17] (version 3.4) in R. The raw counts were converted to log2-counts-per-million (logCPM) followed by linear modeling and the mean–variance relationship was modeled with precision weights using the voom [18] method in limma. The differential expression analysis (tumor vs. control) was performed using *t*-tests implemented in the limma package [19]. The P-values were adjusted using the Benjamini and Hochberg method. The cut-off values were FDR < 0.05 and |log2 FC| > 2 for DEGs and FDR < 0.05 and |log2 FC| > 1.5 for DEMs.

### Functional enrichment analysis

For the identified DEGs, gene ontology (GO) and Kyoto Encyclopedia of Genes and Genomes (KEGG) pathway enrichment analyses were performed using multifaceted analysis tool for human transcriptome (MATHT) (www.biocloudservice.com). The GO analysis included biological process, cellular component, and molecular function categories. P < 0.05 was used as a threshold for the identification of significant GO terms and pathways.

### PPI network analysis

Based on the PPIs in the search tool for the retrieval of interacting genes/proteins (STRING, http://string-db.org/) [20], a PPI analysis of the DEGs was performed with threshold required confidence (combined score) of > 0.4. The obtained PPI pairs were then visualized to construct a PPI network using Cytoscape (http://www.cytoscape.org/) [21]. The topological properties of nodes in the PPI network were analyzed using CytoNCA [22] plugin (version 2.1.6, http://apps.cytoscape.org/apps/cytonca). Based on estimates of degree centrality, betweenness centrality, and closeness centrality of nodes, key nodes involved in the PPI network, named hub proteins, were identified [23].

### miRNA–mRNA regulatory network analysis

The target genes of DEMs were predicted using the miRWalk 2.0 tool [24, 25] (http://zmf.umm.uni-heidelberg.de/apps/zmf/mirwalk2/). The prediction criterion was that the target gene must be identified in three or more of the following prediction databases: miRWalk (http://mirwalk.uni-hd.de/), miRanda (http://www.microrna.org/miranda.html), RNA22 (http://cm.jefferson.edu/rna22v1.0/), and Targetscan (http://www.targetscan.org). Then, target genes were combined with DEGs to select DEM-differentially expressed target gene pairs. Subsequently, the Pearson correlation coefficients were calculated for DEM-differentially expressed target genes pairs and pairs with *r* < − 0.4 (negative regulation) were used to construct the miRNA-mRNA negative regulatory network. The key miRNAs and genes were identified based on a network topological property analysis.

### miRNA function analysis

For genes involved in the miRNA-mRNA negative regulatory network, GO and KEGG pathway enrichment analyses were performed using clusterProfiler [26] in R. These GO terms and KEGG pathways were interpreted as functions of miRNAs involved in the miRNA-mRNA regulatory network.

### Associations between miRNAs/mRNAs and tumor stage

According to the clinical stage and metastasis information obtained from TCGA, samples were divided into the following groups: stage III–IV vs. stage I–II and M1 vs. M0. Differential analyses of stage III–IV vs. stage I–II and M1 vs. M0 were performed to identify DEMs and DEMs associated with stage and metastasis status following the same methods described above “Differential expression analysis”. The cut-off values for DEG selection were FDR < 0.05 and |log2 FC| > 0.585, and for DEM selection were FDR < 0.05 and |log2 FC| > 1.

### Survival analysis for DEGs and DEMs

Clinical information related to prognosis, including overall survival and vital status, were obtained. For all DEMs and DEGs, samples were divided into high and low miRNA/mRNA expression groups based on the median expression values. Kaplan–Meier (KM) curves were then generated using survminer (https://cran.r-project.org/web/packages/survminer/index.html) in R and were analyzed using the log-rank test with a significance threshold of P < 0.05.

### Verification of the expression levels of key mRNAs and miRNAs

An additional nine PTC tumor samples (P6–P14) and paired peritumoral normal thyroid tissues (N6–N14) were collected for qRT-PCR verification of the expression levels of key mRNAs and miRNAs. The detailed patient information is listed in Table 1. Total RNA was extracted from tissue samples using TRIzol reagent (9109; TaKaRa, Dalian, China). cDNA was generated with PrimeScript™ RT Master Mix (RR036A; TaKaRa) and the PrimeScript™ II 1st Strand cDNA Synthesis Kit (6210A; TaKaRa). PCR amplification was performed using the ABI ViiA7 real-time PCR system (Applied Biosystems, Foster City, CA, USA) using Power SYBR Green PCR Master (4367659; Thermo, Waltham, MA, USA). *GAPDH* and *U6* were used as endogenous controls. The primers used for in the study are listed in Table 1. The relative gene expression levels were determined with the 2^−ΔΔCT^ method.

### Statistical analysis

Data are expressed as mean ± standard error of the mean (SEM). Differences between groups were analyzed using Student’s *t*-test implemented in SPSS 22.0 (SPSS, Inc., Chicago, IL, USA). P < 0.05 was considered statistically significant. Experiments were repeated three times.

## Results

### DEG and DEM identification

**Fig. 1:**
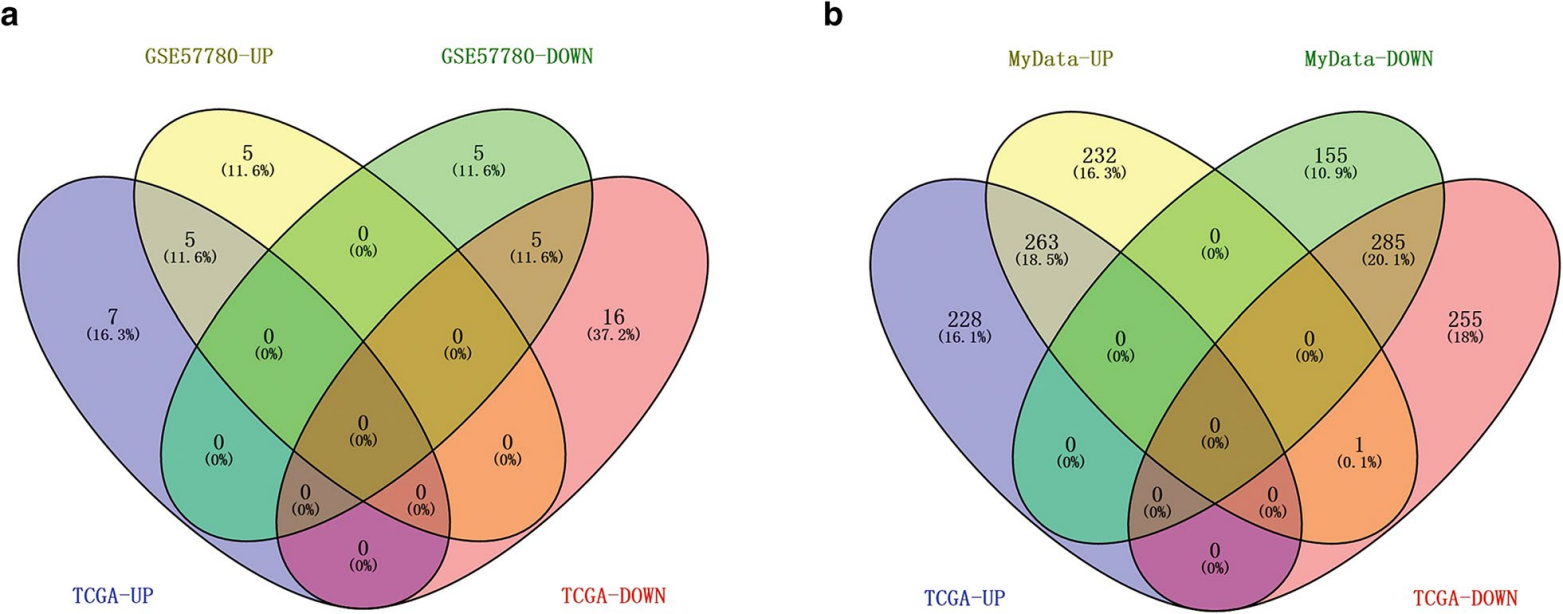
Venn diagram of differentially expressed miRNAs (**a**) and genes (**b**)

A total of 496 up-regulated and 440 down-regulated DEGs were identified using our own mRNA-seq data. For publicly available datasets, 12 up-regulated and 21 down-regulated DEMs were identified using TCGA miRNA-seq data, 491 up-regulated and 541 down-regulated DEGs were identified using TCGA mRNA-seq data, and 14 up-regulated and 15 down-regulated DEGs were identified using GSE57780 data. Subsequently, we constructed a Venn diagram for the DEGs and DEMs. As shown in Fig. 1, 10 intersecting DEMs (5 up- and 5 down-regulation), such as miR-222, miR-221, miR-146b, and miR-21 (Fig. 1a), and 548 intersecting DEGs, including 263 up- and 285 down-regulated DEGs, were identified (Fig. 1b).

### Functional enrichment analysis

**Fig. 2:**
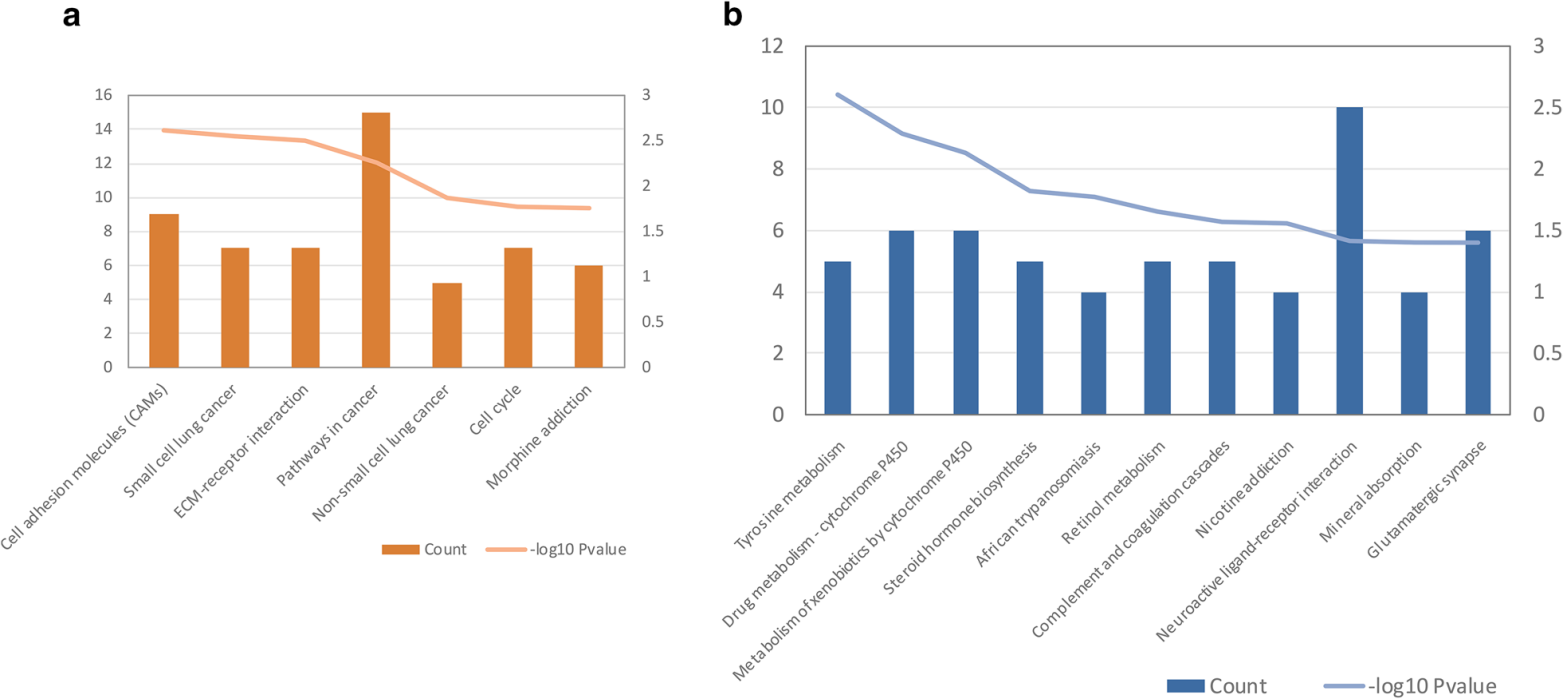
Pathways enriched for the intersecting up-regulated differentially expressed genes (**a**) and down-regulated differentially expressed genes (**b**). Left-hand *Y*-axis indicates the number of genes for each term; right-hand *Y*-axis indicates the − log10 P-value

A functional enrichment analysis of the intersecting DEGs revealed that the up-regulated DEGs were significantly involved in cell adhesion, signal transduction, cell cycle, and cancer-related GO terms and pathways (Table 2 and Fig. 2a). The down-regulated DEGs were significantly enriched in bicarbonate transport, negative regulation of growth, tyrosine metabolism, and drug metabolism-associated GO terms and pathways (Table 2 and Fig. 2b).

**Table 2:** Enriched gene ontology (GO) terms for intersecting up-regulated differentially expressed genes (A) and down-regulated differentially expressed genes (B) (top 5)

Term	Count	P-value
A: up-regulated genes		
GO:0007155~cell adhesion	23	3.99E−07
GO:0030198~extracellular matrix organization	15	5.63E−07
GO:0007165~signal transduction	37	3.65E−06
GO:0030574~collagen catabolic process	8	2.72E−05
GO:0001525~angiogenesis	13	6.29E−05
B: down-regulated genes		
GO:0015701~bicarbonate transport	7	3.85E−05
GO:0045926~negative regulation of growth	5	1.40E−04
GO:0007586~digestion	7	2.94E−04
GO:0015671~oxygen transport	4	0.001197
GO:0006898~receptor-mediated endocytosis	10	0.001578

### PPI network analysis

**Fig. 3:**
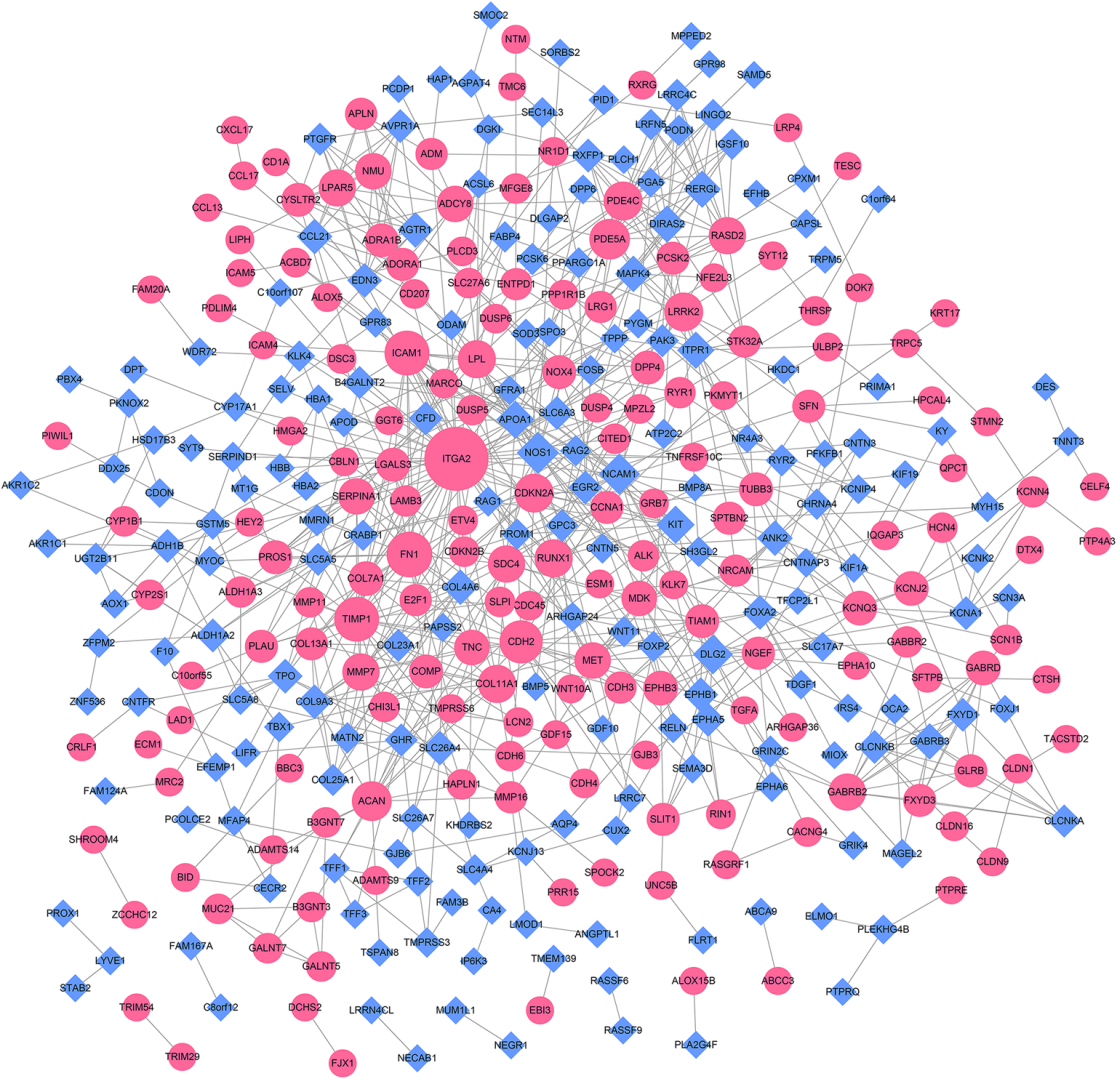
Protein–protein interaction network of differentially expressed genes (DEGs). Red circles represent up-regulated DEGs and each blue rhombus represents a down-regulated DEG. The node size represents the connectivity degree

The PPI network included 369 nodes and 841 interaction pairs (Fig. 3). A topological property analysis of nodes in the PPI network showed that integrin subunit alpha 2 (*ITGA2*), intercellular adhesion molecule 1 (*ICAM1*), fibronectin 1 (*FN1*), tissue inhibitor of metalloproteinases 1 (*TIMP1*), nitric oxide synthase 1 (*NOS1*), cadherin 2 (*CDH2*), and neural cell adhesion molecule 1 (*NCAM1*) had high scores, among other loci. The top 10 nodes with high topological property scores are shown in Table 3.

**Table 3:** Topological property scores for nodes in the protein–protein interaction network (top 10)

Gene	Degree	Gene	Betweenness	Gene	Closeness
ITGA2	44	ITGA2	36,376.34	ITGA2	0.033948
FN1	22	NOS1	16,422.47	NOS1	0.033666
ICAM1	21	CDH2	10,276.65	ICAM1	0.033653
TIMP1	21	NCAM1	9752.948	NCAM1	0.03362
CDH2	19	ICAM1	9480.07	KIT	0.0335
NOS1	18	TIMP1	9079.515	TIMP1	0.033488
NCAM1	17	DLG2	8711.626	APOA1	0.033473
PDE5A	16	APOA1	7099.04	FN1	0.033455
DLG2	15	ACAN	6705.147	MET	0.033439
ACAN	15	PDE5A	6650.764	PDE5A	0.033379

### miRNA-mRNA regulatory network analysis

**Fig. 4:**
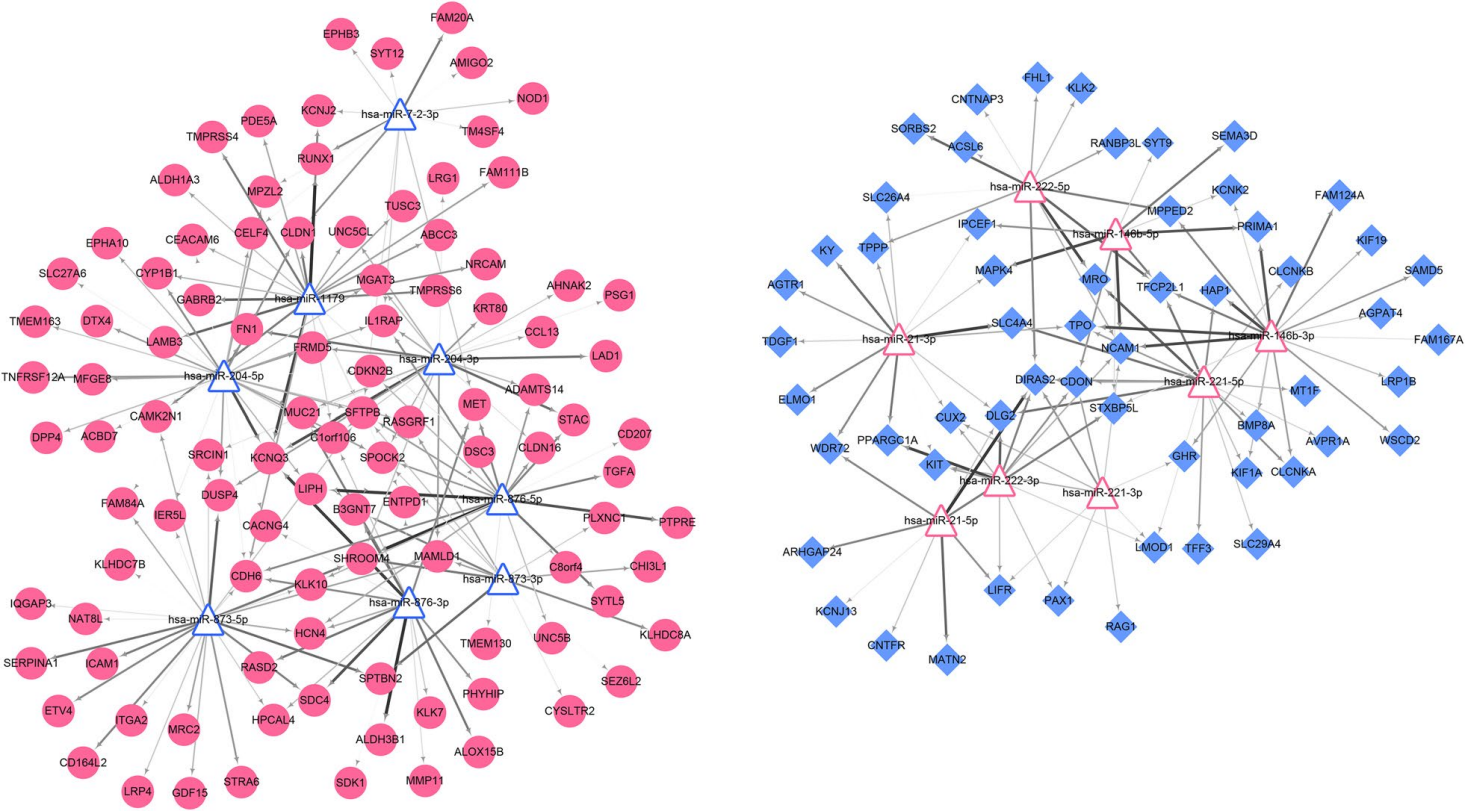
miRNA-mRNA negative regulatory networks of differentially expressed genes (DEGs) and miRNAs (DEMs). Each red circle represents an up-regulated DEG and each blue rhombus represents a down-regulated DEG; red triangles represent up-regulated DEMs and blue triangles represent down-regulated DEMs. Line thicknesses represents the Pearson correlation coefficient

The constructed miRNA–mRNA negatively regulatory networks are shown in Fig. 4. The network included 175 nodes (102 up-regulated mRNAs, 57 down-regulated mRNAs, 8 up-regulated miRNAs, and 8 down-regulated mRNAs) and 272 interacting pairs. A topological property analysis showed that miR-204-5p regulated the largest number of target genes (n = 31), such as such as TNF receptor superfamily member 12A (*TNFRSF12A*), and surfactant protein B (*SFTPB*) was regulated by 6 miRNAs (the most one), such as miR-204-5p.

### miRNA function analysis

**Fig. 5:**
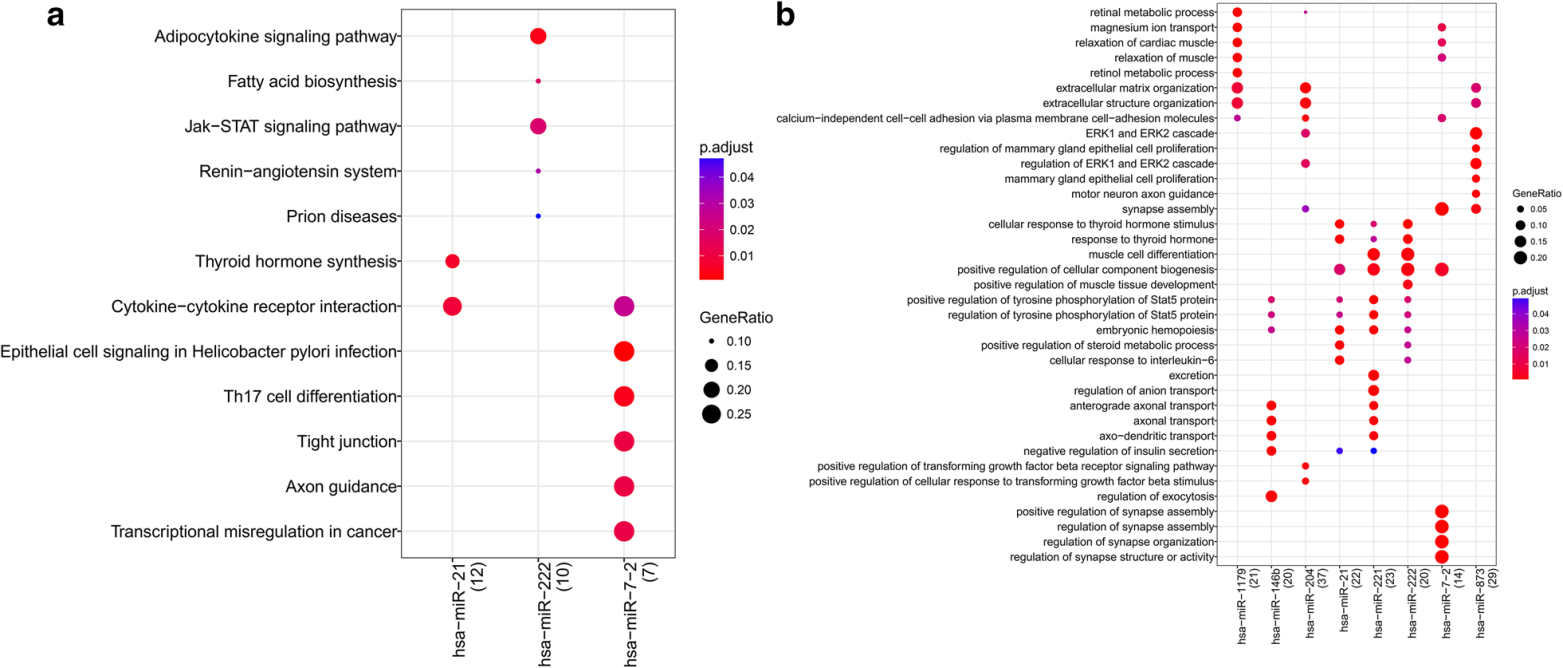
Functional analysis of differentially expressed genes miRNAs (DEMs) in miRNA–mRNA negative regulatory networks. **a** Encyclopedia of genes and genomes (KEGG) pathway; **b** gene ontology (GO) term

Based on the target genes of DEMs involved in the miRNA–mRNA negative regulatory networks, we investigated GO function and pathway enrichment for these DEMs. As shown in Fig. 5, miR-21 was associated with thyroid hormone synthesis and cytokine–cytokine receptor interactions; miR-222 was involved in the adipocytokine signaling pathway and Jak-STAT signaling pathway; miR-7-2 was associated with cytokine–cytokine receptor interactions and tight junctions.

### Associations between miRNAs/mRNAs and tumor stage

Applying the thresholds indicated in “Methods”, 4 DEMs, i.e., miR-146b, miR-204, miR-1179, and miR-7-2, and 61 DEGs, such as *FN1*, baculoviral IAP repeat containing 7 (*BIRC7*), and matrix metallopeptidase 7 (*MMP7*), were identified for the comparison between stage III–IV and stage I–II (Table 4). Additionally, the expression trends of these miRNAs/mRNAs were in accord with those identified in tumor vs. normal tissues. Neither DEMs nor DEGs were identified in the M1 vs. M0 comparison.

**Table 4:** miRNAs/mRNAs associated with tumor stage

Type	Up-regulated	Down-regulated
miRNA	hsa-mir-146b	hsa-mir-204, hsa-mir-1179, hsa-mir-7-2
mRNA	EPHA10, FAM178B, BIRC7, FN1, SLPI, SYT12, ADCY8, B3GNT3, CXCL17, KLK7, IGFL2, SLC34A2, PDZK1IP1, MMP7, TMPRSS4, PSG1, CEACAM6, CD207, SFTPB, CHI3L1, TMPRSS6, CD1A, CCL17, MUC21, COL11A1	CA4, PKHD1L1, ZNF536, ZMAT4, TPO, TFF3, EDN3, KIF1A, NWD1, LRP1B, KIF19, CHRNA4, DIO1, MRO, SLC5A8, MT1G, ZNF804B, HS6ST3, FLRT1, IRS4, DPP6, SLC26A7, OCA2, COL9A3, FOXJ1, CUX2, STXBP5L, WSCD2, SELV, TBX22, GRIK4, KCNJ13, CLCNKA, LINGO2, FER1L6, PKNOX2

### Survival analysis of DEGs and DEMs

**Fig. 6:**
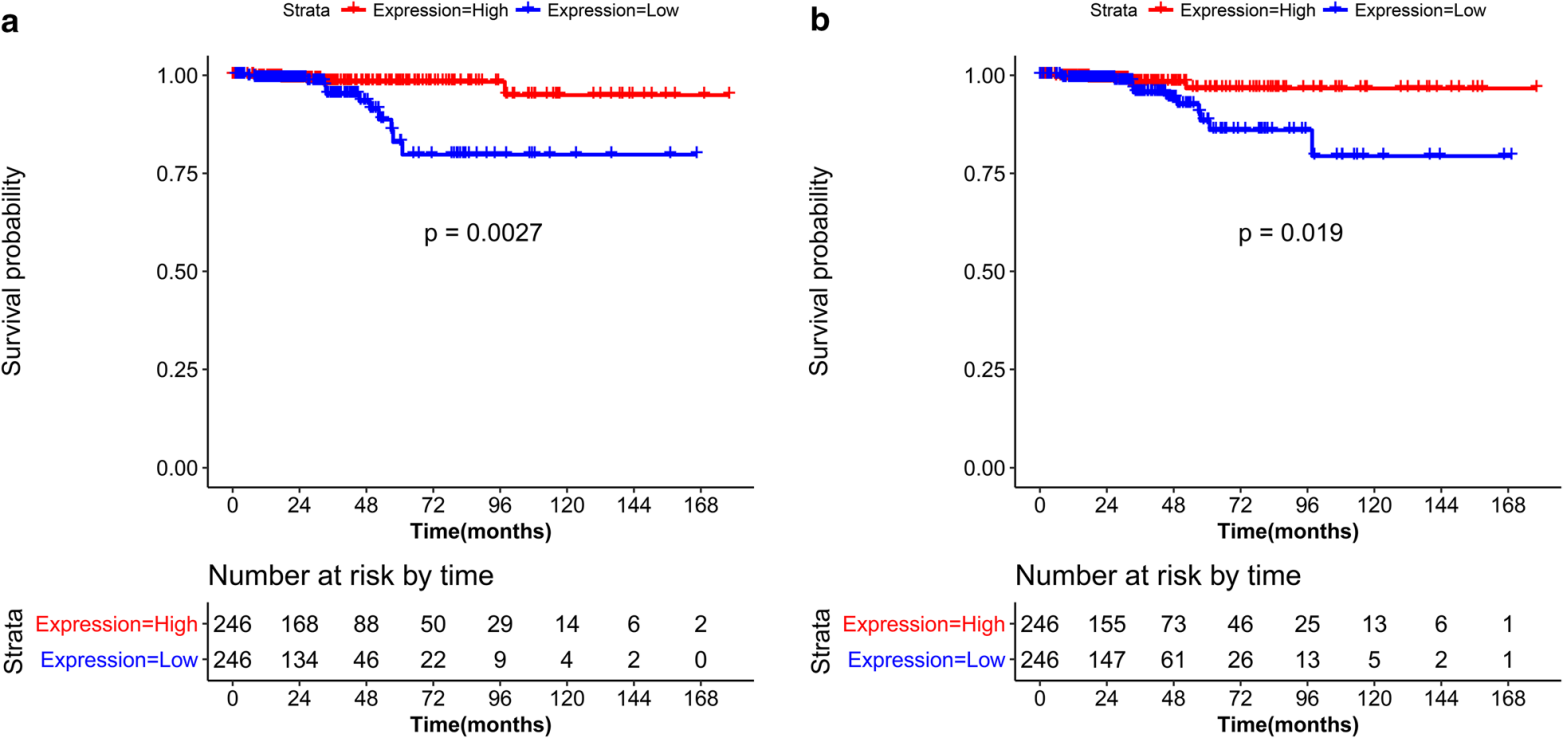
Prognosis-associated Kaplan–Meier (KM) curves for *TNFRSF12A* (**a**) and *CLDN1* (**b**)

Using overall survival and vital status information, we performed a survival analysis for DEGs and DEMs. A total of 59 DEGs were associated with prognosis, such as *TNFRSF12A* (Fig. 6a) and claudin 1 (*CLDN1*) (Fig. 6b), while no prognosis-associated DEMs were identified. Based on the comparation of the 61 tumor stage and 59 prognosis related DEGs, Kallikrein-related peptidase 7 (KLK7) and KLK 10 were presented in both sets. The differences in survival probabilities between the high and low gene expression groups were significant (P < 0.05).

### Expression levels of key mRNAs and miRNAs

**Fig. 7:**
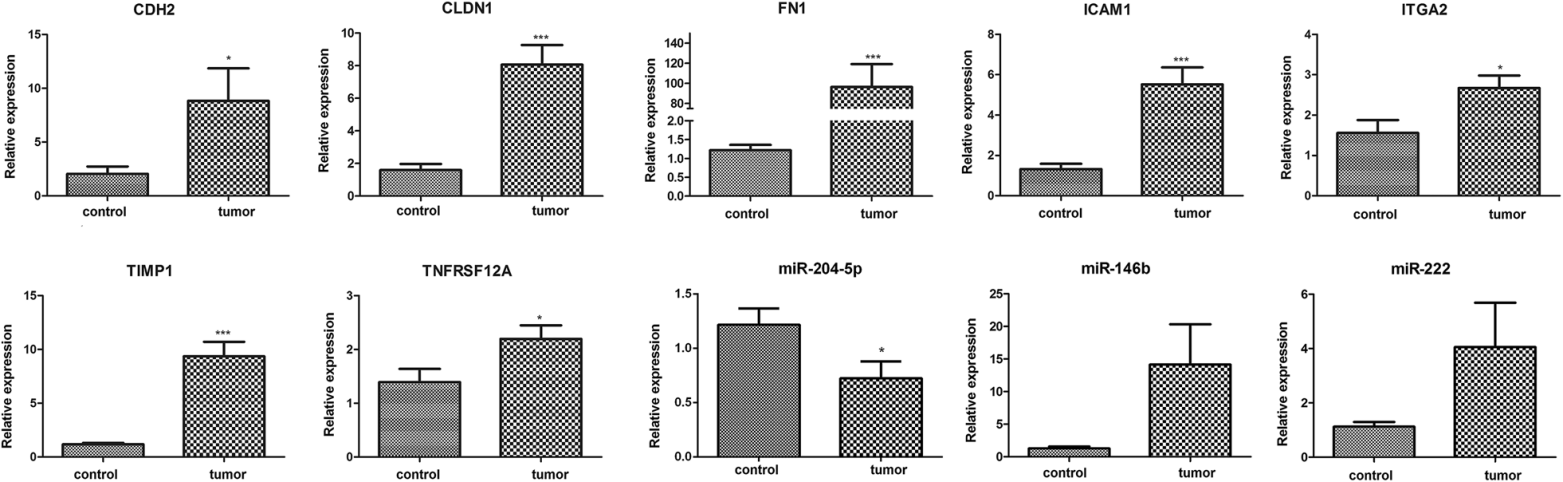
The relative expression levels of seven mRNAs (*ITGA2*, *FN1*, *ICAM1*, *CDH2*, *TIMP1*, *CLDN1*, and *TNFRSF12A*) and three miRNAs (miR-204-5p, miR-146b, and miR-222) in tumor samples and paired peritumoral thyroid tissue samples (control). *P < 0.05, ***P < 0.01 compared with control

The expression levels of seven mRNAs (*ITGA2*, *FN1*, *ICAM1*, *CDH2*, *TIMP1*, *CLDN1*, and *TNFRSF12A*) and three miRNAs (miR-204-5p, miR-146b, and miR-222) were evaluated by qRT-PCR. As shown in Fig. 7, in comparison with the control group, except for miR-204-5p, the expression levels of *ITGA2*, *FN1*, *ICAM1*, *CDH2*, *TIMP1*, *CLDN1*, *TNFRSF12A*, miR-146b, and miR-222 were all up-regulated in the tumor group, consistent with the results of the bioinformatics analyses. Additionally, significant differences were identified in miR-204-5p, *ITGA2*, *FN1*, *ICAM1*, *CDH2*, *TIMP1*, *CLDN1*, and *TNFRSF12A* expression between groups (P < 0.05 or 0.01).

## Discussion

In the present study, numerous DEGs and DEMs between tumor and control groups were identified in four datasets. In a Venn diagram analysis, 10 intersecting DEMs, like miR-204-5p, miR-7-2, miR-146b, and miR-222, and 548 intersecting DEGs were identified. The intersecting DEGs were significantly enriched in cell adhesion and cancer-related GO terms and pathways. In the constructed PPI network, ITGA2, FN1, ICAM1, TIMP1 and CDH2 were identified as hub proteins. In the miRNA–mRNA negative regulatory networks, miR-204-5p regulated the largest number of target genes. Additionally, miR-146b, miR-204, miR-7-2, and *FN1* were associated with the tumor stage of PTC, and *TNFRSF12A* and *CLDN1* were related to the prognosis of PTC.

Cell adhesion determines cell polarity and is involved in tissue maintenance. Abnormalities in cell adhesion are a morphological hallmark of malignant tumors, and have effects on the biological characteristics of cancers [27]. The present results showed that up-regulated DEGs identified in multiple datasets, such as *ICAM1* and *CDH2*, were significantly involved in cell adhesion-associated functions and pathways (GO: 0007155~cell adhesion, hsa04514: cell adhesion molecules). Specifically, ICAM1 and CDH2 were hub proteins in the PPI network. ICAM1, a transmembrane glycoprotein receptor, belongs to the immunoglobulin superfamily of adhesion molecules [28]. The expression of ICAM1 is elevated in many human cancers, including PTC [29]. ICAM1 can facilitate the spread of metastatic cancer cells to secondary sites by recruiting inflammatory cells that release angiogenic and growth factors stimulating angiogenesis, cell proliferation, and invasion [30]. CDH2 belongs to the cadherin gene family of cell–cell adhesion molecules and encodes N-cadherin. CDH2 is commonly up-regulated in various human cancers, including thyroid cancer [31], and its overexpression can promote tumor cell invasion [32]. In addition, CDH2 plays a significant role in epithelial-mesenchymal transition, a pivotal event for tumor cell acquisition of metastatic ability [33]. Importantly, *ICAM1* and *CDH2* were up-regulated in PTC tissues in a validation experiment. Taken together, the pathway and genes associated with cell adhesion may play important roles in PTC development.

Most complex networks, including PPI networks, are scale-free, containing a small number of highly connected nodes (hubs) and a large number of poorly connected nodes (non-hubs). A genome-wide analysis has shown that the deletion of a hub is more likely to be lethal than the deletion of a non-hub, suggesting the importance of hubs in network organization [23]. In this study, we identified several hub proteins in the PPI network. In addition to ICAM1 and CDH2 mentioned above, TIMP1, ITGA2 and FN1 had high degrees and accordingly may play important roles in PTC. Additionally, *FN1* was identified to be a clinical stage-associated gene. ITGA2 is an important collagen receptor on epithelial cells, and its expression regulated during normal cell differentiation and altered during tumorigenesis [34]. FN1 is an extracellular matrix protein synthesized by fibroblasts, and is often associated with goiters [35]. *FN1* was first reported to be overexpressed in thyroid cancer in 1988 [36]. It is the most strikingly up-regulated marker in intermediate-risk PTC and is considered a negative prognostic marker in patients with PTC [37]. In the present study, *TIMP1*, *ITGA2* and *FN1* were up-regulated in PTC tissues. Additionally, *ITGA2* and *FN1* were associated with GO terms and pathways related to extracellular matrix (ECM) organization and cancer (hsa05222: small cell lung cancer, hsa04512: ECM-receptor interaction, hsa05200: pathways in cancer). ECM is essential at various stages of the carcinogenic process and abnormal ECM is considered a hallmark of cancer [38]. Therefore, *ITGA2* and *FN1* may play key roles in PTC progression via these cancer-related pathways. TIMP1 is recognized as a multifunctional protein. Inhibiting matrix metalloproteinases and antiangiogenic activity are two important roles of TIMP1 [39, 40]. Study has demonstrated inhibition of TIMP1-mediated tumor invasion and metastasis in many types of tumor cells [41, 42]. Elevated serum and tissue levels of TIMP1 have been found in cancer patients [43]. In this study, *TIMP1* was demonstrated to up-regulate in PTC tumor tissue, which was in consistent with the previous study. Thus, TIMP1 may serves as key biomarker of PTC.

miRNAs negatively regulate gene expression at the post-transcriptional level and have profound biological importance. The dysregulation of miRNA expression has been implicated in human cancers [44]. In the present study, several DEMs were identified, such as miR-204-5p, miR-7-2, miR-146b, and miR-222. miR-204-5p was a hub in the miRNA–mRNA regulatory network, regulating the most target genes. It was also associated with clinical stage. A recent study found that miR-204-5p was down-regulated in PTC and acted as a tumor suppressor [45]. In accordance with this finding, miR-204-5p was down-regulated in PTC tissues in our study and regulated *TNFRSF12A*, a prognosis-associated DEG. Besides, result of qRT-PCR also showed that miR-204-5p expression was significantly decreased while *TNFRSF12A* expression was significantly increased in tumor compared with control. Based on a GO functional enrichment analysis, *TNFRSF12A* was related to angiogenesis. A previous study has found that angiogenesis is essential for tumor growth and progression [46]. Importantly, Tanaka et al. [47] reported that the balance between angiogenic and antiangiogenic factors is correlated with the distinct invasion of PTC, suggesting the importance of angiogenesis in PTC.

miR-7-2 was another down-regulated DEM associated with clinical stage. Based on a functional enrichment analysis, the target genes of miR-7-2, such as *CLDN1*, were significantly enriched for hsa04530: tight junction pathway. CLDN1 encodes a member of the claudin family, a component of tight junction strands. Tight junctions are one mode of cell-to-cell adhesion in endothelial or epithelial cell sheets; they can alter cell polarity, cell fate, and cell migration, thereby impacting cancer progression. Alterations in tight junctions facilitate breast cancer initiation and progression [48]. Interestingly, *CLDN1* was a prognosis-associated DEG in this study. Therefore, miR-7-2 and *CLDN1* may be used as biomarkers of stage and prognosis in PTC.

miR-146b and miR-222 were up-regulated in the tumor group in this study, and miR-146b was a DEM associated with clinical stage. miR-146b has been implicated in several cancer types by microarray analyses, such as cervical cancers, breast cancer, and prostate cancers [49]. Moreover, miR-222 is a cancer-related miRNA and has been reported to promote cancer cell proliferation [50]. Importantly, miR-146b and miR-222 are overexpressed in PTC tissue and are significantly associated with extrathyroidal invasion [51]. Validation experiment also found that miR-146b and miR-222 were up-regulated in PTC tissue, although there was no significant difference. Therefore, our data were consistent with previous results, further supporting the important role of miR-146b and miR-222 in PTC.

By comparing the 61 tumor stage and 59 prognosis related DEGs, there were two members in the intersection: KLK7 and KLK 10. Kallikrein are serine proteases which have many physiological functions, containing the remodeling of ECM. They are able to function individually or in cascade pathway(s) and acted as diagnostic and prognostic biomarkers for tumors [52]. The expression of KLK7 and KLK10 in PTC were significantly upregulated, with a mean fold change of 28.8 and 27.8, reletive to matched normal tissue, suggesting that KLK7 and KLK10 might be useful biomarker for the diagnosis of PTC [53]. In this study, KLK7 and KLK10 were identified as the biomarkers of tumor stage and prognosis of PTC, which were consistent with the previous report. In addition, miR-1179 was another DEM related to tumor stage of PTC. A recently report showed that miR-1179 inhibited glioblastoma cell proliferation through targeting E2F transcription factor 5 [54]. In this study, miR-1179 was down-regulated in PTC patients, and the regulation mechanism of miR-1179 in the PTC progress was need further study. Although some genes and miRNAs markers were identified and validated, this study had some limitations. The sample size was small; therefore, we will collect additional samples in the future to further investigate the roles of these genes and miRNAs in the pathogenesis of PTC.

In conclusion, our results suggested the important roles of *ITGA2*, *FN1*, *ICAM1*, *TIMP1* and *CDH2* in the progression of PTC via various cancer-related pathways. Additionally, miR-204-5p, miR-7-2, miR-146b, and miR-222 may play critical roles in tumorigenesis of PTC. Furthermore, *FN1*, miR-204-5p, miR-7-2, and miR-146b may be used as biomarkers for PTC staging, and *CLDN1* and *TNFRSF12A* may serve as prognostic markers.
